# Exploration of solar radiation data from three geo-political zones in Nigeria

**DOI:** 10.1016/j.dib.2017.05.017

**Published:** 2017-05-17

**Authors:** Adebowale O. Adejumo, Esivue A. Suleiman, Hilary I. Okagbue

**Affiliations:** a Department of Mathematics, Covenant University, Ota, Nigeria; b Department of Statistics, University of Ilorin, Ilorin, Nigeria

**Keywords:** ANOVA, Solar radiation, Tukey׳s Post Hoc, Port Harcourt, Sokoto, Ibadan

## Abstract

In this paper, readings of solar radiation received at three meteorological sites in Nigeria were analysed. Analysis of Variance (ANOVA) statistical test was carried out on the data set to observe the significant differences on radiations for each quarter of the specified years. The data were obtained in raw form from Nigerian Meteorological Agency (NIMET), Oshodi, Lagos. In order to get a clear description and visualization of the fluctuations of the radiation data, each year were considered independently, where it was discovered that for the 3rd quarter of each year, there is a great fall in the intensity of the solar radiation to as low as 73.27 (W/m^2^), 101.66 (W/m^2^), 158.51 (W/m^2^) for Ibadan, Port-Harcourt and Sokoto respectively. A detailed data description is available for the averages across months for each quarter. The data can provide insights on the health implications of exposure to solar radiation and the effect of solar radiation on climate change, food production, rainfall and flood patterns.

**Specification Table**

**Table:** 

**Subject area**	Environmental Science
**More specific subject area**	Solar Radiation
**Type of data**	Table and figure
**How data was acquired**	Unprocessed secondary data
**Data format**	Processed as Monthly Averages Across Quarters from 2011 to 2015 for Three Meteorological Sites
**Experimental factors**	Data obtained from Nigerian Meteorological Agency (NIMET)
**Experimental features**	Computational Analysis: Analysis of Variance (ANOVA) with Post Hoc Test and Correlation Analysis.
**Data source location**	Ibadan, Port-Harcourt and Sokoto Meteorological Stations.
**Data accessibility**	All the data are in this data article.
**Software**	Microsoft Excel and Minitab 17 Statistical Software

**Value of the data**

● The energy sector of the economy can incorporate the data set and findings for the utilization of solar radiation received from the sites.
● The vitality of these data set is widely recognised in the energy research community for forecasting minutely, hourly, daily and monthly solar radiations using time series tools which could also cater for volatility that exist in the data.
● For educational purposes and environmental studies. See similar works [1], [2], [3], [4], [5], [6], [7], [8], [9], [10], [11], [12], [13], [14], [15], [16], [17], [18], [19], [20], [21], [22], [23], [24], [25], [26], [27], [28], [29], [30], [31], [32], [33].
● Findings from the data bring the awareness of the Nigerian government to the most suitable location for the establishment of both solar plants and research institutes to generate electricity.
● The data can provide insights on the health implications of exposure to solar radiation.

## 1 Data

The raw data for this work were obtained from Nigerian Meteorological Agency (NIMET) Oshodi Lagos, as daily averages on solar radiation for three weather stations namely; Ibadan, Sokoto and Port-Harcourt. The readings were taken using the Gunn-Bellani Radiation Integrator measuring the radiations in millilitres (ml). However, for the sake of this research, the readings were converted to Watts per Sq. meters (1 ml to 13.153 W/m^2^) covering from 1st of January, 2011 to 31st of December, 2015 and further transformed into monthly-quarterly averages (Table 7) for the specified years using Microsoft Excel software.

Table 1a is the statistical summary of the quarterly averages for solar radiation from the 1st of January 2011 to 31st of December 2015. Meanwhile, it was observed that on an average, Sokoto receives the highest intensity of solar radiation followed by Port Harcourt and Ibadan.

**Table 1a:** Summary for the quarterly average for the sites.

**Variable**	**N**	**Mean**	**S.E Mean**	**Std. Dev.**	**Minimum**	**Q1**	**Median**	**Q3**	**Maximum**
**Ibadan**	60	142.24	3.84	29.76	73.27	115.07	151.91	166.75	186.77
**Sokoto**	60	235.01	4.40	34.08	158.51	217.05	237.96	258.57	308.72
**Port H**	60	153.29	3.85	29.85	101.66	129.08	156.36	179.17	231.96

Furthermore, Table 1b shows that the data set from all sites exhibit negative kurtosis and skew, implying that the distributions are light-tailed and skewed to the left respectively.

**Table 1b:** Summary for the quarterly average for the sites.

**Variable**	**Kurtosis**	**Skewness**
**Ibadan**	−0.77	−0.60
**Sokoto**	−0.02	−0.45
**Port H**	−0.57	−0.05

**Fig. 1:**
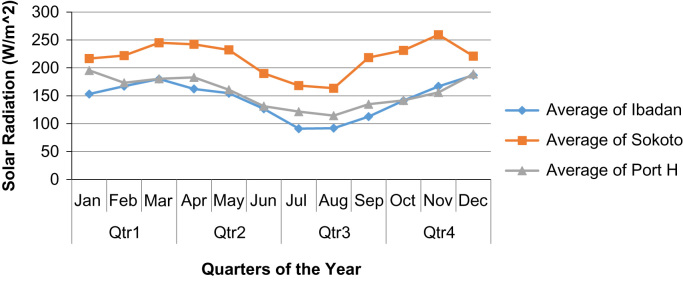
Quarterly averages of solar radiation for the three sites in the year 2011.

**Fig. 2:**
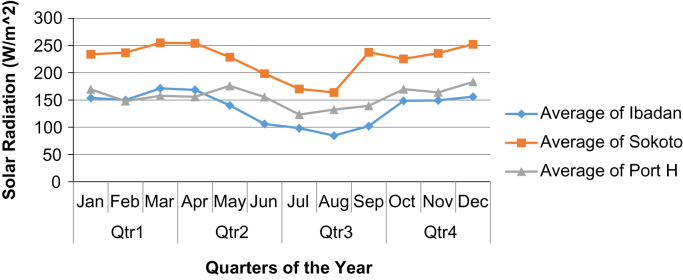
Quarterly averages of solar radiation for the three sites in the year 2012.

**Fig. 3:**
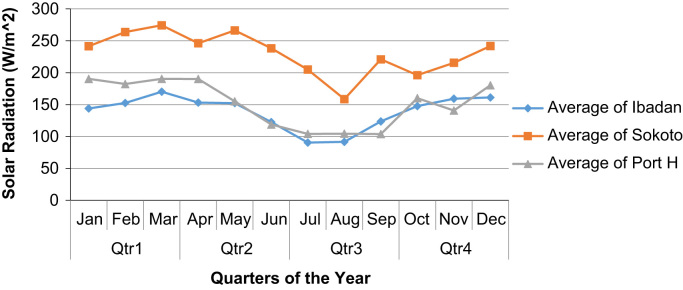
Quarterly averages of solar radiation for the three sites in the year 2013.

**Fig. 4:**
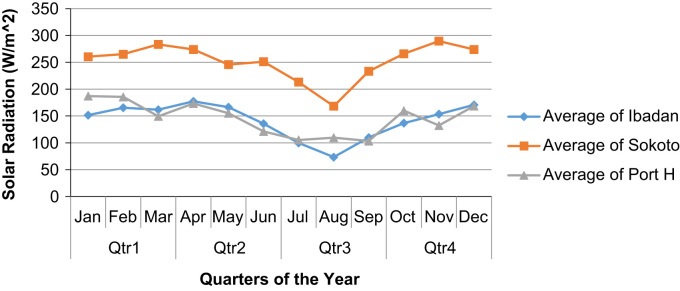
Quarterly averages of solar radiation for the three sites in the year 2014.

**Fig. 5:**
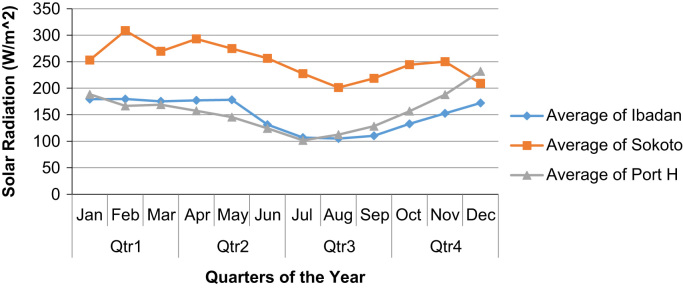
Quarterly averages of solar radiation for the three sites in the year 2015.

From the graphs (Fig. 1, Fig. 2, Fig. 3, Fig. 4, Fig. 5), it was observed that Sokoto was top on the presented charts with an exception on December 2015. This validates that the closer the earth׳s surface is to the sun, the greater the radiations it receives, which is well applicable to the case of Sokoto ranking top among the other stations with a height of 309 m above sea level. Furthermore, the solar radiation received at Sokoto increases yearly within the 1st quarter. It has to be noted that the *y*-axis of the figures is the solar radiation reading for the zones measured in Watt per square meter.

## 2 Methods and materials

The summary of the location sites of the raw data are displayed in Table 2.

**Table 2:** Location of the sites.

**Sites**	**Latitude**	**Longitude**	**Height (m)**
**Ibadan**	07.22′	03.59′	224.01
**Sokoto**	12.55′	05.12′	309.0
**Port Harcourt**	05.01′	06.57′	247.0

Linear correlation is traditionally used to roughly determine the relationship between two variables. Table 3 shows the correlation matrix between the three meteorological stations. Though independent, the correlations among each station are positive and that of Sokoto–Ibadan and Ibadan–Port Harcourt are highly positively correlated. It is either the solar radiation levels are increasing or decreasing at the three sites simultaneously.

**Table 3:** 3×3 Correlation matrix for the sites.

***Sites***	***Ibadan***	***Sokoto***	***Port Harcourt***
**Ibadan**	1		
**Sokoto**	0.741099	1	
**Port H**	0.755118	0.426821562	1

The ANOVA test carried out on the data set for all sites and the result was displayed in Table 4, Table 5, Table 6. The results showed significant differences in the means for solar radiation received quarterly at the stations independently.

**Table 4:** Analysis of Variance (ANOVA) for Port Harcourt.

**Source of variation**	**D.F**	**S.S**	**M.S**	***F*****-value**	***P*****-value**
**Quarters**	3	31672	10557.3	28.29	0.000
**Error**	56	20898	373.2		
**Total**	59	52570

**Table 5:** Analysis of Variance (ANOVA) for Sokoto.

**Source of variation**	**D.F**	**S.S**	**M.S**	***F*****-value**	***P*****-value**
**Quarters**	3	29161	9720.4	13.83	0.000
**Error**	56	39364	702.9		
**Total**	59	68526

**Table 6:** Analysis of Variance (ANOVA) for Ibadan.

**Source of variation**	**D.F**	**S.S**	**M.S**	***F*****-value**	***P*****-value**
**Quarters**	3	38059	12686.3	50.04	0.000
**Error**	56	14197	253.5		
**Total**	59	52256			

The significant differences in the means as revealed from the ANOVA results led to further analysis using the Tukey׳s Simultaneous 95% Confidence Interval Post Hoc test. The aim is to detect the specific quarters where differences lie across the specified years.

**Fig. 6:**
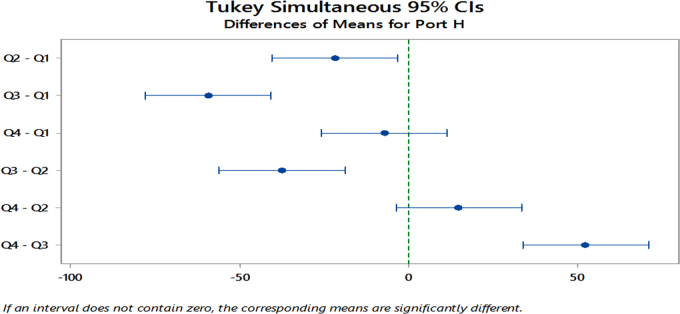
Tukey׳s Post Hoc test for mean-quarterly differences in Port Harcourt site from 2011 to 2015.

**Fig. 7:**
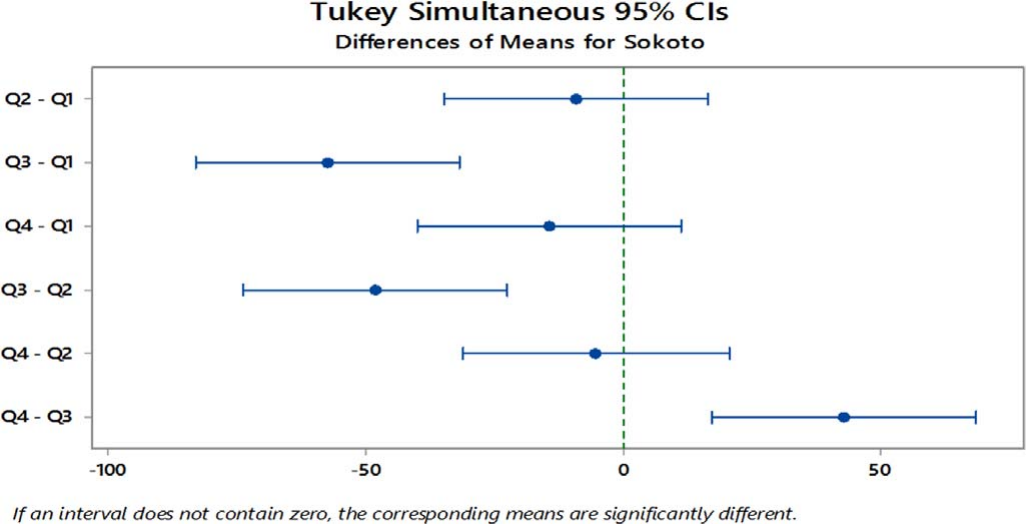
Tukey׳s Post Hoc test for mean-quarterly differences in Sokoto site from 2011 to 2015.

**Fig. 8:**
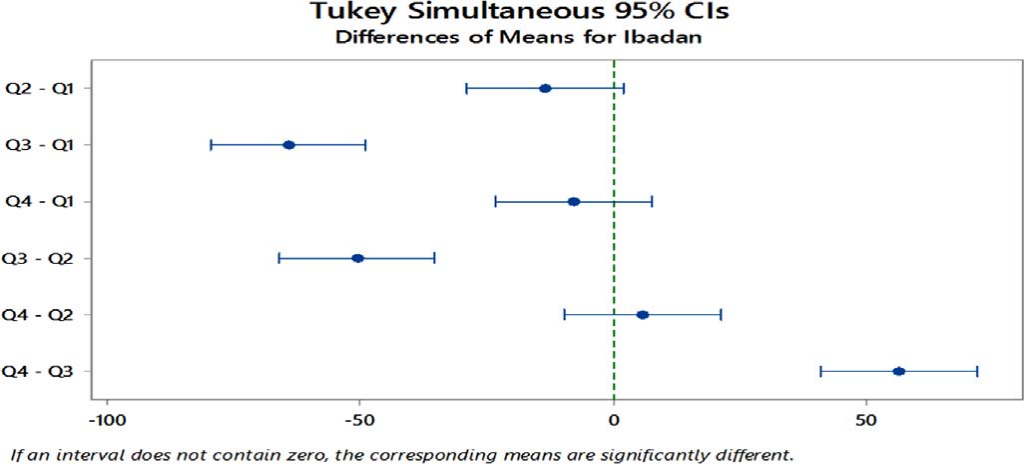
Tukey׳s Post Hoc test for mean-quarterly differences in Ibadan site from 2011 to 2015.

The results revealed that for Port Harcourt, significant differences lie within all other quarters except for the 1st and 4th quarters and for the 2nd and 4th quarters yearly as seen in Fig. 6. Similarly, Fig. 7 shows that for Sokoto, the 1st and 3rd quarters, 3rd and 4th quarters and the 2nd and 3rd quarters as having significant differences. Lastly, Fig. 8 shows that for Ibadan, significance differences exists between the 1st and 3rd Quarters, 2nd and 3rd Quarters and the 3rd and 4th Quarters for these years. Minitab17 software was implemented for analysis on the solar radiation data, which produced the ANOVA results and the Post Hoc test for the stations (Table 7).

**Table 7:** Monthly-quarterly solar radiation for three sites from 2011 to 2015.

**Year**	**Month**	**Quarter**	**Ibadan**	**Sokoto**	**Port Harcourt**
**2011**	Jan	Q1	152.9544	216.6395	195.3404
**2011**	Feb	Q1	167.1816	221.9066	173.1943
**2011**	Mar	Q1	179.9389	244.9392	180.448
**2011**	Apr	Q2	162.218	242.0993	182.9117
**2011**	May	Q2	154.397	232.1683	160.7612
**2011**	Jun	Q2	126.53	189.8827	131.3527
**2011**	Jul	Q3	90.88166	168.1862	121.4725
**2011**	Aug	Q3	91.89994	163.3494	114.3021
**2011**	Sep	Q3	112.588	218.2928	134.7724
**2011**	Oct	Q4	141.0745	231.2348	141.5836
**2011**	Nov	Q4	166.8653	259.2857	155.8608
**2011**	Dec	Q4	186.7699	220.9247	188.9337
**2012**	Jan	Q1	153.8454	233.9503	169.7561
**2012**	Feb	Q1	149.9874	236.8866	148.0371
**2012**	Mar	Q1	171.4956	255.0796	157.5473
**2012**	Apr	Q2	168.6628	254.1561	155.8032
**2012**	May	Q2	140.2683	228.6467	175.9798
**2012**	Jun	Q2	106.2308	198.5635	155.5539
**2012**	Jul	Q3	98.30663	170.3925	123.297
**2012**	Aug	Q3	84.72954	163.9434	132.4191
**2012**	Sep	Q3	102.2412	237.7151	139.4198
**2012**	Oct	Q4	148.6692	225.507	169.9258
**2012**	Nov	Q4	149.4159	235.8298	164.1032
**2012**	Dec	Q4	156.1366	252.3218	183.3756
**2013**	Jan	Q1	143.9172	241.5025	190.2066
**2013**	Feb	Q1	152.4786	263.7138	182.1664
**2013**	Mar	Q1	170.1379	274.2572	190.2914
**2013**	Apr	Q2	153.0987	246.2206	190.1896
**2013**	May	Q2	152.318	266.2807	155.0758
**2013**	Jun	Q2	122.4965	238.1973	118.5945
**2013**	Jul	Q3	90.37251	204.8868	104.1193
**2013**	Aug	Q3	91.51808	158.5125	104.4163
**2013**	Sep	Q3	123.5487	220.8356	103.7318
**2013**	Oct	Q4	147.5236	196.0405	160.1248
**2013**	Nov	Q4	159.2367	215.5635	140.6912
**2013**	Dec	Q4	161.1007	241.7571	180.2359
**2014**	Jan	Q1	151.5119	260.3407	187.0669
**2014**	Feb	Q1	165.4435	265.0291	185.4546
**2014**	Mar	Q1	161.6947	283.3369	149.3904
**2014**	Apr	Q2	177.256	273.8853	173.354
**2014**	May	Q2	166.4042	245.7454	155.1183
**2014**	Jun	Q2	135.6931	251.1309	121.3127
**2014**	Jul	Q3	99.66434	213.1179	105.4346
**2014**	Aug	Q3	73.27386	168.3135	109.7623
**2014**	Sep	Q3	109.8259	233.287	103.2934
**2014**	Oct	Q4	136.8316	265.9048	159.7854
**2014**	Nov	Q4	153.5371	289.4494	132.2734
**2014**	Dec	Q4	170.6895	273.8075	168.4408
**2015**	Jan	Q1	179.5146	253.1279	188.5943
**2015**	Feb	Q1	179.7237	308.7152	166.6179
**2015**	Mar	Q1	175.399	269.7174	169.0348
**2015**	Apr	Q2	177.2122	293.1761	157.4829
**2015**	May	Q2	178.2418	274.8512	145.487
**2015**	Jun	Q2	131.3527	256.4359	124.2063
**2015**	Jul	Q3	106.962	227.5436	101.6585
**2015**	Aug	Q3	105.1376	201.4077	112.5626
**2015**	Sep	Q3	110.3959	218.6873	128.3276
**2015**	Oct	Q4	132.8434	244.4301	156.8578
**2015**	Nov	Q4	152.7041	250.2541	187.9975
**2015**	Dec	Q4	172.3018	209.2145	231.9561
